# TMS Correlates of Pyramidal Tract Signs and Clinical Motor Status in Patients with Cervical Spondylotic Myelopathy

**DOI:** 10.3390/brainsci10110806

**Published:** 2020-10-31

**Authors:** Giuseppe Lanza, Valentina Puglisi, Luisa Vinciguerra, Francesco Fisicaro, Carla Vagli, Mariagiovanna Cantone, Giovanni Pennisi, Manuela Pennisi, Rita Bella

**Affiliations:** 1 Department of Surgery and Medical-Surgical Specialties, University of Catania, Via Santa Sofia, 78-95123 Catania, Italy; pennigi@unict.it; 2 Department of Neurology IC, Oasi Research Institute–IRCCS, Via Conte Ruggero, 73-94018 Troina, Italy; 3 Department of Neurology and Stroke Unit, ASST Cremona, Viale Concordia, 1-26100 Cremona, Italy; valentina.puglisi@asst-cremona.it (V.P.); luisa.vinciguerra@asst-cremona.it (L.V.); 4 Department of Biomedical and Biotechnological Sciences, University of Catania, Via Santa Sofia, 89-95123 Catania, Italy; drfrancescofisicaro@gmail.com (F.F.); manuela.pennisi@unict.it (M.P.); 5 Department of Neurology, San Giovanni di Dio Hospital, ASP Agrigento, Contrada Consolida, 92100 Agrigento, Italy; carlavagli@gmail.com; 6 Department of Neurology, Sant’Elia Hospital, ASP Caltanissetta, Via Luigi Russo, 6-93100 Caltanissetta, Italy; m.cantone@asp.cl.it; 7 Department of Medical and Surgical Sciences and Advanced Technologies, University of Catania, Via Santa Sofia, 78-95123 Catania, Italy; rbella@unict.it

**Keywords:** motor-evoked potentials, transcranial magnetic stimulation, corticospinal conduction, pyramidal signs, motor status, degenerative cervical myelopathy, cervical spondylotic myelopathy, clinical neuroscience

## Abstract

Background: While the association between motor-evoked potential (MEP) abnormalities and motor deficit is well established, few studies have reported the correlation between MEPs and signs of pyramidal tract dysfunction without motor weakness. We assessed MEPs in patients with pyramidal signs, including motor deficits, compared to patients with pyramidal signs but without weakness. Methods: Forty-three patients with cervical spondylotic myelopathy (CSM) were dichotomized into 21 with pyramidal signs including motor deficit (Group 1) and 22 with pyramidal signs and normal strength (Group 2), and both groups were compared to 33 healthy controls (Group 0). MEPs were bilaterally recorded from the first dorsal interosseous and tibialis anterior muscle. The central motor conduction time (CMCT) was estimated as the difference between MEP latency and peripheral latency by magnetic stimulation. Peak-to-peak MEP amplitude and right-to-left differences were also measured. Results: Participants were age-, sex-, and height-matched. MEP latency in four limbs and CMCT in the lower limbs were prolonged, and MEP amplitude in the lower limbs decreased in Group 1 compared to the others. Unlike motor deficit, pyramidal signs were not associated with MEP measures, even when considering age, sex, and height as confounding factors. Conclusions: In CSM, isolated pyramidal signs may not be associated, at this stage, with MEP changes.

## 1. Introduction

Motor cortex excitability and corticospinal conduction can be noninvasively probed in vivo and in “real time” by recording motor-evoked potentials (MEPs) in response to transcranial magnetic stimulation (TMS) [1]. In brief, TMS generates a rapid and high-intensity electromagnetic field able to basically pass unattenuated through the scalp. When the pulses stimulate the primary motor cortex (M1), the electromagnetic induction activates the excitable cells which, in turn, transmit the pulses along the corticospinal tract and the peripheral nerves to the muscles. Using conventional electromyography (EMG) surface electrodes, MEPs from a contralateral skeletal muscle can be easily obtained [2]. On the basis of these principles, MEP recording allows for the objective, reproducible, and painless assessment of the central motor pathways, including the activation status of facilitatory and inhibitory neuronal networks [3] and interhemispheric functioning [4].

Since it was first introduced [5], diagnostic TMS has been applied in almost all neurological disorders and systemic diseases with neurological involvement [6], showing a high sensitivity in the detection of corticospinal tract lesions [7] and also allowing preclinical diagnosis [8,9,10] and disease monitoring [11]. In particular, MEP latency and central motor conduction time (CMCT) are viewed as measures of cortical–spinal myelination, whereas MEP amplitude is viewed as an index of the integrity of the neuronal axons from motor cortical areas to spinal motoneurons [2]. As well as being routinely used as a diagnostic tool to study the central motor pathways and to support, quantify, and monitor a central motor deficit [2], TMS in research settings is employed to map motor and cognitive functions, explore neural networks, and modulate brain activity for therapeutic purposes [12,13,14,15,16].

Although TMS is considered to be a sensitive examination, electrophysiological techniques are known to be susceptible to several sources of variability [17,18,19]. In this context, studies on the TMS correlates of clinical motor status have been reported in patients with motor deficits resulting from different underlying causes and of varying degrees, ranging from a very mild paresis to a complete palsy [17]. For instance, the association between certain MEP measures, particularly CMCT, and loss of power was previously described in multiple sclerosis, in which the longer the CMCT, the weaker the force [20]. In stroke patients, cortical MEP findings showed a good correlation both radiologically with the lesion and clinically with the motor deficit [21]. Namely, the degree of motor weakness was significantly correlated with the severity of cortical MEP findings but not with deep tendon reflex and the Babinski sign [21]. However, the study included both ischemic and hemorrhagic strokes of different locations and severities. Moreover, the lack of correlation between MEP findings and deep tendon reflex and the Babinski sign was not discussed, and there are no reproductions in other disease models. Lastly, unlike earlier studies [20,21], the role of TMS in defining the value of signs of pyramidal tract dysfunction in cases other than motor deficits—such as hyperactivity of deep tendon reflexes, Hoffman and/or Babinski sign, clonus, and spasticity—is still undefined. Indeed, while the diagnostic role of MEPs is unequivocal when pyramidal signs are associated with motor deficit, even of very mild severity, no TMS study to date has been carried out in patients with signs of pyramidal tract dysfunction without any clinical weakness, the occurrence of which is very common in clinical practice, thus precluding a deeper understanding of their clinical significance and diagnostic impact.

In this scenario, although signs of pyramidal tract dysfunction have been classically considered to be the consequence of a single pathophysiological mechanism, i.e., a cortical–spinal tract lesion, recent evidence suggests that they might be different phenomena [22]. For instance, spasticity is one of the most common signs in humans with spinal cord injury (SCI); however, its mechanisms of action remain poorly understood. The different contribution of the corticospinal and reticulospinal tract in the control of a spastic muscle was recently demonstrated using TMS in patients with chronic incomplete SCI. In particular, patients showed smaller MEPs over the leg area of the M1, reduced maximal voluntary contraction, and larger reticulospinal gain compared with participants with no or low spasticity and healthy controls [22].

In the present study, we aimed at evaluating routine MEPs in four limbs in patients with pyramidal signs, including motor deficit, compared to those with pyramidal signs and normal muscle strength, and both were compared to healthy controls. For this purpose, the model of cervical spondylotic myelopathy (CSM) was considered. Unlike motor disorders caused by other central nervous system diseases (e.g., stroke, multiple sclerosis, motor neuron disease), the clinical and radiological picture of patients with CSM is rather homogeneous and the motor signs and symptoms follow a myotomal distribution [23]. Moreover, the usefulness of TMS in the diagnosis and management of CSM is widely documented, since MEPs can supplement clinical and imaging findings of the spinal level and severity, help with a differential diagnosis of other conditions, and allow for the quantification of motor function before and after surgery and rehabilitation [24,25]. We hypothesize that pyramidal signs without motor deficit might not be sufficient to determine significant changes to basic TMS measures.

## 2. Materials and Methods

### 2.1. Participants and Assessment

A total of 43 right-handed patients with CSM were consecutively recruited from the TMS Lab of the “Azienda Ospedaliera Universitaria Policlinico Gaspare Rodolico-San Marco” of Catania (Italy), from November 2018 to October 2019. CSM, clinically suspected by trained neurologists, was confirmed using 1.5 T-magnetic resonance imaging (MRI).

Only patients with a definite diagnosis were included, whereas those with doubtful clinical presentations, an incomplete diagnostic workup, or cervical cord compression without myelopathy were excluded. Subjects were also excluded if they had any of the following: other forms of degenerative cervical myelopathy, such as those caused by ossification of the posterior longitudinal ligament, ossification of the ligamentum flavum, or degenerative disc diseases; other neurological diseases (e.g., traumatic head or back injury, stroke or chronic cerebrovascular disease, degenerative, inflammatory, or demyelinating disorders, tumors, etc.); peripheral neuropathies, radiculopathies, or neuromuscular disorders; previous cranial or spinal surgery; major psychiatric diseases; acute, advanced, or chronic unstable medical illnesses (including diabetes, hypothyroidism, and neoplasm); alcohol or drug abuse; current treatment with neuroactive drugs or any other medication able to affect cortical excitability [26,27,28]; a history or presence of epilepsy; implanted electrical biomedical devices (i.e., pacemaker, prosthesis); pregnancy at the time of testing; any contraindication to TMS [2].

All patients underwent a demographic and clinical assessment and a detailed neurological examination before TMS. The demographic assessment included age, sex, and height. Special attention was paid to the evaluation of signs of pyramidal tract dysfunction, including hyperactivity of deep tendon reflexes, Hoffman and/or Babinski sign, clonus, and spasticity. Since brisk tendon reflexes may even be observed in normal persons, this was considered as a pyramidal sign when there was a significant reflex asymmetry and/or a spread of the tendon reflex outside the stimulated territory [17]. The motor assessment system used to clinically quantify the motor deficit is the widely known grading system of the Medical Research Council (MRC), which ranges from 0 (complete palsy) to 5 (normal power) [29]. Given that different muscle groups were examined for each arm and leg, bilaterally, a cumulative motor score, i.e., that provided by the American Spinal Injury Association (ASIA) scale, was used, thus providing a global assessment of the motor weakness. On the basis of a neurological examination, participants were dichotomized into patients with pyramidal signs including motor deficit patients (Group 1) and patients with pyramidal signs without any clinical weakness (Group 2), and patients of both groups were compared with right-handed healthy controls (Group 0). The presence of signs of pyramidal tract dysfunction were considered as a single whole category without further differentiation based on the specific sign.

This study was carried out in accordance with the recommendations of the International Federation of Clinical Neurophysiology for the diagnostic use of TMS [2]. All subjects provided their informed consent for inclusion prior to participation in the study. The study was conducted in accordance with the Declaration of Helsinki, and the protocol was approved by the Ethics Committee of the “Azienda Ospedaliera Universitaria Policlinico Gaspare Rodolico-San Marco” of Catania (Italy) (approval code: 9/2018/PO).

### 2.2. Transcranial Magnetic Stimulation

A high-power MagStim 220 monopulse stimulator (The Magstim Co., Ltd., Whitland, Dyfed, UK) connected to a 90-mm circular coil with an inner diameter of 5 cm was employed. Since the round coil stimulates a larger cortical volume, the positioning over the target region is easier than it is with the focal, “figure-of-eight”-shaped coil. The circular coil also results in better depth penetration, which is advantageous for TMS of the M1 leg area. Finally, the round coil is less susceptible to the unavoidable minimal changes in coil position [2,30].

As in a routine diagnostic exam, the patient was seated on an armchair while recordings from the distal limb muscles were taken. Standard silver/silver chloride cup surface (EMG)electrodes (9-mm diameter), which were filled with jelly and applied over the first dorsal interosseous (FDI) and tibialis anterior (TA) muscles in a conventional belly-tendon montage, were used for MEP recordings from the contralateral side of stimulation [2]. The handle of the coil pointed backward, whereas the coil center was positioned tangentially over the Cz (according to the international electroencephalography 10–20 system) for recordings from the FDI muscle and over the Fz for recordings from the TA muscle [30].

For TMS of the right hemisphere, the current direction within the circular coil was clockwise such that the induced cortical current was perpendicular to the cortex in a posterior–anterior direction, and vice versa for the left hemisphere, as recommended [31]. After the location was identified, the coil position and orientation were slightly adapted until the best excitation point (“hot spot”) was targeted, especially for the localization of the lower limb cortical area. Once the position was defined, the outer rim of the coil was marked with a soft-tip demographic pen on the scalp to enable the examiner to maintain a constant position.

An MEP in the relaxed muscle was recorded first. After that, according to the abovementioned guidelines [2], five MEPs were obtained while patients produced a transient tonic muscular activity, and the shortest MEP latency was used for CMCT estimation. Similarly, given that a routine TMS exam evaluates the transcranially induced motor response with the biggest amplitude, the MEP with the largest peak-to-peak size was considered. To ensure MEP reproducibility, especially in patients with mild motor deficit, a strain gauge was used to achieve a valid and reproducible muscle activation, which was about 10%–20% of the subject’s maximum voluntary contraction. TMS of the motor cortex was performed at the same time as muscular contraction in order to avoid muscle preactivation. Preactivation of the muscles, indeed, can cause variations in the EMG background activity between patients, and even within the same subject [2].

To determine the peripheral motor latency, motor nerve root stimulation was performed by applying the coil center on the 7th cervical and the 4th lumbar spinous process for upper and lower limbs, respectively. To ensure reliability, three reproducible peripheral motor responses were recorded at rest and averaged. Thereafter, the peripheral motor latency from the cervical or lumbar magnetic stimulation was subtracted from the MEP cortical latency to obtain the CMCT. Therefore, the time of conduction from neurons within the motor cortex to those within the anterior horn of the spinal cord defines the CMCT, thus reflecting the motor conduction time from the upper to the lower motor neuron [2].

Given that the resting stimulation threshold for a 2.0 T-magnetic stimulator (as used in this study) is approximately 50%–55% for upper limbs and 60%–65% for lower limbs of the maximal output [32,33,34], motor responses were all obtained at 80% of the maximum stimulator output. In such a way, a visible contraction of the target muscle was observed after each stimulation. We also verified that MEP cortical latency did not reduce further, nor did the amplitude increase further when the intensity was increased to over 80% [30]. The amplification and filtering (bandwidth 3–3000 Hz) of the motor responses, as well as the offline analysis, were carried out using a 2-channel Medelec Synergy system (Oxford Instruments Medical, Inc., Surrey, UK).

### 2.3. Statistical Analysis

Statistical software (SPSS, version 22.0) was used for all analyses. The variables analyzed for each study group (Group 1: patients with pyramidal signs including motor deficit; Group 2: patients with pyramidal signs without any clinical weakness; Group 0: healthy controls) included age, sex, height, disease duration, ASIA score, and TMS measures (MEP amplitude, MEP latency, and CMCT from the FDI and TA muscle, recorded bilaterally). Since the Kolmogorov–Smirnov test indicated that most target variables were not normally distributed, a nonparametric analysis was carried out. Data were presented as median (I–III quartile), or number and percentage, as appropriate. The three groups were compared by an intergroup analysis (0 vs. 1 vs. 2) using the Kruskal–Wallis test for skewed continuous data (age, height, TMS parameters) and the chi-square test for the categorical variable (sex), followed by post hoc analysis. The intragroup right-to-left difference of each TMS parameter was determined for each group (0, 1, and 2) using the Mann–Whitney test. Groups 1 and 2 were compared for disease duration and ASIA score with the Mann–Whitney test.

Multiple linear regression analysis of TMS parameters (dependent variables) and the influence of physical (age, sex, and body height) and clinical variables (pyramidal signs and motor deficit) as predictors were computed in all groups. We applied a backward elimination stepwise procedure for the choice of the best predictive variables in accordance with the Akaike information criterion. A 95% confidence level was set with a 5% alpha error. Statistical significance was set at *p* < 0.05.

## 3. Results

Group 1 included 21 patients with pyramidal signs including motor deficit (median age 57.0 years, interquartile range 14.0; 66.7% males), Group 2 included 22 patients with pyramidal signs without any clinical weakness (median age 49.5 years, interquartile range 21.8; 36.4% males), and Group 0 included 33 healthy controls (median age 54.0 years, interquartile range 22.5; 54.5% males). None of the patients had any relevant co-morbidities and had a normal brain MRI.

Group 1 exhibited mild tetraparesis (ASIA upper-extremity motor score: median 40/50, interquartile range 1; lower-extremity motor score: median 36/50, interquartile range 7), without a significant side distribution or symptom distribution, and had pyramidal signs in four limbs (each demonstrated brisk tendon reflexes and spreading of the reflex stimulation territory, most had bilateral Hoffman and/or Babinski sign, a few had clonus, and none had spasticity). Group 2 showed a similar distribution of the pyramidal signs, but without any clinically detectable motor deficit (MRC 5 in all muscular districts). Therefore, none of the patients had unilateral pyramidal signs. Group 0 (healthy controls) did not have any pyramidal sign or motor deficit (MRC 5 in all muscular districts).

**Figure 1:**
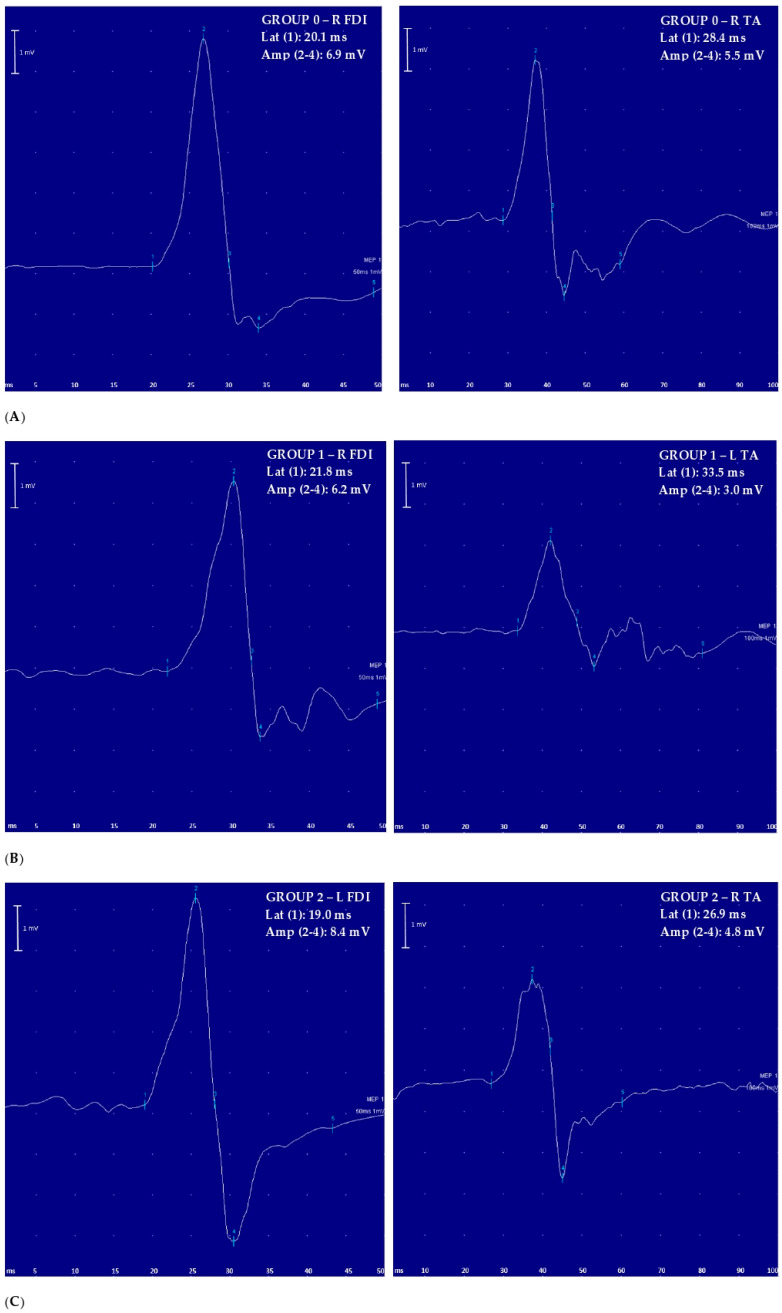
Examples of motor-evoked potential (MEP) recorded for each study group (left side: upper limb; right side: lower limb): (**A**) Group 0: healthy controls; (**B**) Group 1: patients with signs of pyramidal tract dysfunction including motor deficit; (**C**) Group 2: patients with signs of pyramidal tract dysfunction without motor deficit. Amp = MEP amplitude; FDI = first dorsal interosseous muscle; L = left; Lat = MEP latency; MEP = motor-evoked potential; R = right; TA = tibialis anterior muscle; 50 ms = temporal resolution of the screen (sweep) for upper limb recordings; 100 ms = temporal resolution of the screen (sweep) for lower limb recordings; 1 mV = amplification factor of the screen; numbers in bold = values significantly different to control at the group level.

**Figure 2:**
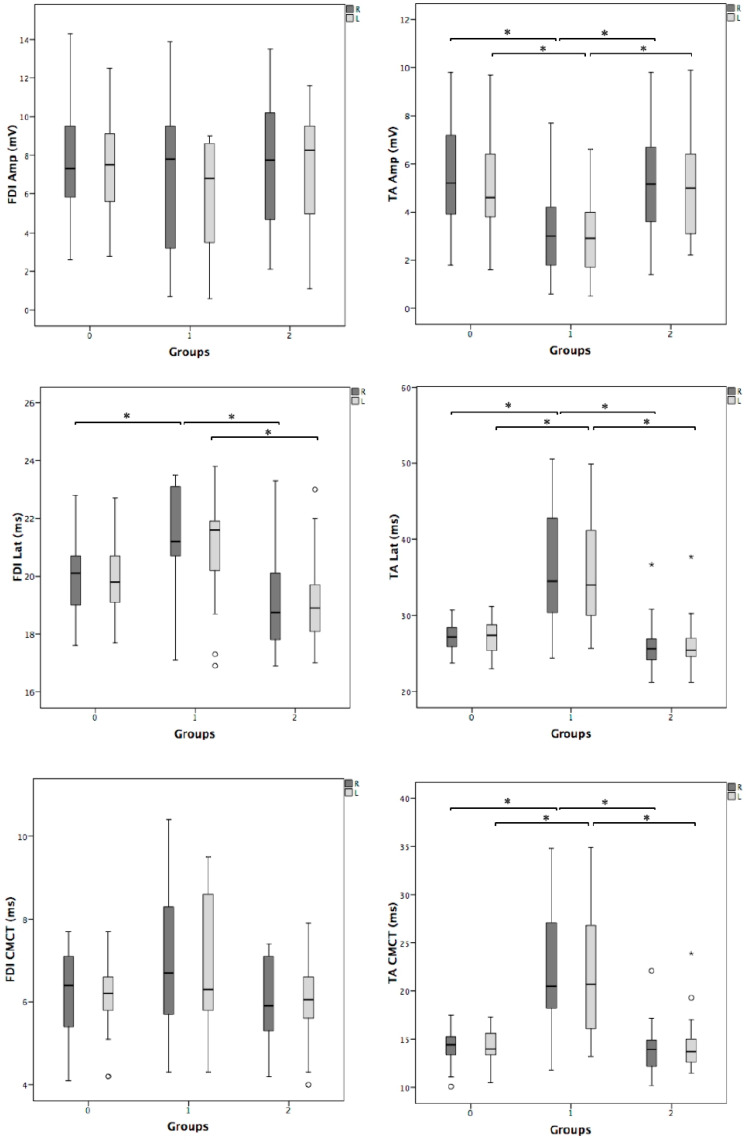
Box-plots of the motor-evoked potentials (MEPs): intergroup analysis (0 vs. 1 vs. 2). Group 0: healthy controls; Group 1: patients with signs of pyramidal tract dysfunction including motor deficit; Group 2: patients with signs of pyramidal tract dysfunction without motor deficit. Amp = MEP amplitude; CMCT = central motor conduction time; FDI = first dorsal interosseous muscle; L = left; Lat = MEP latency; R = right; TA = tibialis anterior muscle; whiskers = interquartile range; * = *p* < 0.05.

Overall, TMS was well tolerated and no significant side effects or discomfort were reported during or after the exam. Figure 1 shows examples of MEP recordings for each study group. Table 1 shows that the three groups were matched for age, sex, body height, and disease duration; as expected, Group 1 exhibited motor weakness in four limbs compared to the other group of patients. As shown in Figure 2, MEP latency in the four limbs and the CMCT in the lower limbs were significantly prolonged in Group 1 with respect to the other two groups. Additionally, compared to the same groups, MEP amplitude from the TA muscle, recorded bilaterally, was significantly decreased in Group 1. No difference was found between Group 2 and 0, and all the other measures, including the right-to-left differences (Table 2), did not differ among the groups.

Linear regression analysis of TMS parameters as dependent variables and clinical variables (pyramidal signs and motor deficit) as predictors showed that motor deficit, but not pyramidal signs, was significantly and independently associated with MEPs (Table 3A). When age, sex, and height were added as predictors to clinical variables (Table 3B), we found that pyramidal signs were not significantly and independently associated with any TMS parameter. Conversely, age, sex, and motor deficit had an independent and significant association with all the TMS measures, except for the MEP amplitude from the left FDI. In other words, according to the multiple linear regression analysis, changes in pyramidal signs were not statistically associated with changes in TMS parameters, also when confounding factors were included (age, sex, and body height).

## 4. Discussion

### 4.1. Main Findings

To the best of our knowledge, this is the first study to directly compare TMS in a homogeneous group of CSM patients with and without motor deficit with healthy controls and correlate pyramidal signs with MEP changes. Abnormal TMS findings, consistent with axonal damage and demyelination, were found in Group 1, thus confirming the clinical and radiological findings of these patients and the diagnostic accuracy of MEPs in CSM patients with even mild motor deficits. More interestingly, however, we observed comparable MEPs between Group 0 and 2, suggesting that isolated pyramidal signs may be interpreted as neurophysiologically “mute” unless associated with other clinical findings. The multiple linear regression analysis supported this view by showing that, unlike motor deficit, pyramidal signs were not associated with any of the TMS measures studied here when taking age, sex, and body height into consideration as confounding factors. Indeed, it is known that some physical features, such as age, sex, and height, may affect MEP response and, therefore, need to be taken into account for an accurate and meaningful interpretation [19,35]. For this reason, when assessing MEPs both in a clinical practice and research setting, patients and controls should be matched for age, sex, and height [36], as carried out in the present study. Moreover, since physical features can be predictors of outcome in different neurological disorders (e.g., the upper limb recovery after stroke [37]), the effect of any of these variables should be considered.

Overall, these results need careful interpretation, given the paucity of previous evidence. An earlier study evaluating the diagnostic value of MEPs in a large sample of patients with different neurological disorders, including those with spinal cord disease, revealed an agreement index between electrophysiological and clinical findings of 87%. However, subclinical abnormalities were found in only 12.5% of patients with spinal cord diseases [17]. A reasonable explanation might be that patients with isolated pyramidal signs may only have a partial involvement of the corticospinal tract. Since TMS indexes are thought to represent the conduction of the fastest motor fibers within the corticospinal bundle [38], MEPs would be normal if these fibers were spared (as is supposed in patients with pyramidal signs alone) [21]. This “subthreshold” damage might explain the electrophysiological findings observed in Group 2. Conversely, in Group 1, the motor lesion would be a “suprathreshold” and, therefore, the cortical stimulus would not be able to generate enough excitatory post-synaptic potentials in spinal motoneurons [39]. Nevertheless, the effects of a spinal cord disease on D-wave and I-wave generations, as well as their propagation along the spine and the mechanisms underlying their plasticity, remain uncertain [40].

On the other hand, it is known that a more powerful activation of the spinal motor neurons can be obtained by simply increasing the stimulus intensity, a modality that activates a larger number of corticospinal fibers through convergence on single motoneurons [41,42]. Indeed, in clinical practice, higher magnetic stimulation intensity is helpful to overcome damaged motor pathways, especially in cases where there is not complete paralysis. To rule out this possibility, we verified that MEPs did not change even when we stimulated at intensities higher than 80% of the maximal stimulator output. This demonstrates that the intensity used was sufficiently high to excite the fast-conducting corticospinal neurons [30].

It should be noted that although most of the pyramidal signs are mediated at the spinal level, they are also influenced by the higher centers [43], and supraspinal motor-related networks may be implicated in functional weakness after a cervical spinal cord injury [44,45]. It has been recently demonstrated that corticomotor conduction through the level of the spinal cord injury is basically preserved, and that the topographic origins of this projection in the M1 are normal [46]. This supports previous evidence regarding normal central motor conduction in chronic and stable patients with cervical spinal cord injury [47,48]. In particular, in these patients, the fast-conducting monosynaptic pathway to the target muscles was relatively normal, a finding consistent with anatomical studies showing the presence of spared axons across the lesion [49,50,51].

On the basis of these results and considerations, the MRI, although it remains the method of choice to display the spinal cord, cannot provide functional information. In this context, TMS can be viewed as the functional counterpart of neuroimaging in assessing spinal cord diseases, including the very early stages [25]. MEPs can also monitor disease progression, ascertain progressive forms, and select the responders to surgical interventions and rehabilitation [52].

### 4.2. Clinical Implications

Overall, finding out whether patients with pyramidal signs but without weakness have altered MEP responses might be of relevance in clinical practice. While the occurrence of MEP changes in patients with motor deficit is well documented [7], the same cannot be said for MEPs in patients with isolated pyramidal signs. An explanation for this can be hypothesized. Ideally, pyramidal signs may be divided into two main categories: “irritative” (or “positive”) and “deficitary” (or “negative”). The former (i.e., hyperactive tendon reflexes, spreading of the reflex stimulation territory, clonus, Hoffman and/or Babinski sign) might indicate preclinical damage of the corticospinal tract, possibly compensating for the incipient motor deficit. When this damage exceeds a certain threshold, signs of a deficit (i.e., overt or latent motor deficit) would appear.

This hypothesis, although requiring further validation, would explain both the common occurrence of pyramidal signs in the absence of a clinically detectable motor deficit and the finding of normal MEPs in subjects with isolated pyramidal signs. Notably, in a similar model affecting brain motor function (i.e., ischemic stroke), a strong correlation was found between MEP amplitude at day 1 and both MRC and Barthel index at day 14, regardless of pyramidal signs [53]. This clearly shows that early TMS is a valuable prognostic tool for post-stroke motor recovery and suggests that muscles with these features may benefit from a targeted rehabilitation program [54,55,56,57]. The relationship between motor weakness and MEP alterations was also investigated by Kim and Park [58] in 50 patients with different diseases affecting the central motor pathway; these were mostly cerebral stroke or hemorrhage patients, with cervical spondylosis reported in three cases (but without mention of any associated myelopathy). The authors found that mean MEP latency and mean CMCT were prolonged in patients with motor pathway lesions, with cortical MEP not being elicited in stroke patients with severe motor weakness in the upper limbs (MRC < 2). Moreover, MEP latency and CMCT prolongation correlated with moderate motor weakness (MRC < 4) [58].

### 4.3. Limitations

Some limitations should be acknowledged. First, as is usual in TMS research, there was a relatively small sample size, although the subjects were carefully screened, thus making the sample very homogeneous. Nevertheless, the limited number of participants did not allow further stratifications—e.g., according to those based on different types of pyramidal signs or levels of myelopathy. However, all patients had a CSM above the C7 level and, therefore, a spinal cord disease could be detectable by recording from the FDI and TA muscles. In any case, given that the lesion site might affect MEP sensitivity [59], a multilevel neurophysiological evaluation of patients with spinal cord disease would be helpful for both research and clinical purposes.

Second, CSM may cause not only spinal cord disease but also nerve root compression, although the peripheral nerve conduction velocity was not determined. However, all subjects did not have any clinical sign or history of peripheral nerve pathology. Moreover, as stated, patients with concomitant peripheral neuropathy and/or radiculopathy were preliminarily excluded.

Another caveat is that only patients with mild motor deficit were studied and, as a consequence, we cannot compare these data with those affected by a severe symptomatology. However, these mildly affected patients allowed us to probe the diagnostic sensitivity of TMS more accurately. It still remains to be determined to what extent the findings observed in this population can be applied in patients with motor deficit secondary to cerebral lesions or motor neuron diseases. Indeed, since we only focused on CSM, this study is not representative of the whole spectrum of clinical conditions involving the pyramidal tract. Therefore, the conclusions concerning the association between MEP abnormalities, pyramidal signs, and motor deficit cannot be generalized.

Fourth, for the CMCT calculation, the peripheral conduction time was estimated by direct spinal root stimulation with TMS. This is a suboptimal approach compared with the F-wave method for example, as it can underestimate the peripheral conduction time, although both techniques have pros and cons [2].

Lastly, this study deals with basic MEPs, which represent a routine diagnostic application of TMS. For detailed research purposes, TMS is able to evaluate different parameters, including a number of excitatory/inhibitory intracortical phenomena, although this was beyond the scope of this clinically oriented study.

## 5. Conclusions

In patients with CSM, diagnostic MEPs represent an accurate test even in patients with mild motor deficit, whereas signs of pyramidal tract dysfunction without clinical weakness may not be associated, at this stage, with basic TMS changes. Further TMS studies in different disease models and with longitudinal exams and more extensive measurements are needed for a better understanding of the impact and progression of MEPs in spinal cord diseases and other neurological disorders.

## Figures and Tables

**Table 1:** Demographics and clinical variables of the three groups.

Variable, Unit	Group 0 (*n* = 33)	Group 1 (*n* = 21)	Group 2 (*n* = 22)	*p* Value	Post Hoc Analysis	
					Difference	*p* Value
Age, years	54.00 (22.50)	57.00 (14.00)	49.50 (21.80)	0.290 *	NS	NS
Sex, male (%) female (%)	18.00 (54.50) 15.00 (45.50)	14.00 (66.70) 7.00 (33.30)	8.00 (36.40) 14.00 (63.60)	0.130 ^†^	NS	NS
Height, cm	163.00 (7.00)	166.00 (10.50)	163.00 (7.80)	0.260 *	NS	NS
Disease duration, months	-	19.5 (8.7)	21.0 (9.0)	0.981 ^‡^	-	-
ASIA UER	-	20.0 (1.0)	25.0 (0.0)	**<0.001 ^‡^**	-	-
ASIA UEL	-	20.0 (1.0)	25.0 (0.0)	**<0.001 ^‡^**	-	-
ASIA LER	-	18.0 (5.0)	25.0 (0.0)	**<0.001 ^‡^**	-	-
ASIA LEL	-	19.0 (3.0)	25.0 (0.0)	**<0.001 ^‡^**	-	-

*Legend*: values are expressed as median and interquartile range; ASIA = American Spinal Injury Association scale; LER = lower extremity, right side; LEL = lower extremity, left side; NS = not significant; UEL = upper extremity, left side; UER = upper extremity, right side; * = Kruskal–Wallis test; † = chi-square test; ‡ = Mann–Whitney test; numbers in bold = statistically significant *p* values.

**Table 2:** Intragroup analysis of transcranial magnetic stimulation (TMS) variables (right vs. left) of the three groups (Mann–Whitney test).

Variable, Unit	Group 0 (*n* = 33), *p*	Group 1 (*n* = 21), *p*	Group 2 (*n* = 22), *p*
FDI amplitude, mV	0.91	0.20	0.75
FDI latency, ms	0.67	0.73	0.78
FDI CMCT, ms	0.39	0.96	0.95
TA amplitude, mV	0.56	0.75	0.83
TA latency, ms	0.95	1.00	0.69
TA CMCT, ms	0.81	0.74	0.73

Legend: *n* = number of subjects; FDI = first dorsal interosseous muscle; TA = tibialis anterior muscle; CMCT = central motor conduction time.

**Table 3:** Predictors of TMS parameters: (**A**) linear regression analysis; (**B**) multiple linear regression analysis.

**(A) Dependent Variable**	**Predictor**	**Std Beta**	***p***	**Adjusted *R*^2^**
Right FDI MEP amplitude	Pyramidal signs	−0.04	0.770	0.02
	Motor deficit	−0.05	0.690	
Right FDI MEP latency	Pyramidal signs	−0.18	0.130	0.21
	Motor deficit	0.56	**<0.001**	
Right FDI CMCT	Pyramidal signs	−0.07	0.550	0.10
	Motor deficit	0.39	**0.003**	
Left FDI MEP amplitude	Pyramidal signs	−0.06	0.630	0.32
	Motor deficit	−0.19	0.146	
Left FDI MEP latency	Pyramidal signs	−0.16	0.199	0.18
	Motor deficit	0.51	**<0.001**	
Left FDI CMCT	Pyramidal signs	0.56	0.741	0.10
	Motor deficit	0.62	**0.005**	
Right TA MEP amplitude	Pyramidal signs	−0.06	0.610	0.10
	Motor deficit	−0.32	**0.015**	
Right TA MEP latency	Pyramidal signs	−0.06	0.530	0.37
	Motor deficit	0.65	**<0.001**	
Right TA CMCT	Pyramidal signs	0.02	0.850	0.30
	Motor deficit	0.58	**<0.001**	
Left TA MEP amplitude	Pyramidal signs	0.35	0.790	0.09
	Motor deficit	−0.38	**0.006**	
Left TA MEP latency	Pyramidal signs	−0.04	0.670	0.34
	Motor deficit	0.63	**<0.001**	
Left TA CMCT	Pyramidal signs	0.006	0.959	0.30
	Motor deficit	0.56	**<0.001**	
**(B) Dependent Variable**	**Predictor**	**Std Beta**	***p***	**Adjusted *R*^2^**
Right FDI MEP amplitude	Age	−0.26	**0.040**	0.07
Right FDI MEP latency	Motor deficit	0.50	**<0.001**	0.22
Right FDI CMCT	Motor deficit	0.41	**0.003**	0.07
Left FDI MEP amplitude	Motor deficit	−0.23	0.090	0.10
	Age	−0.23	0.050	
Left FDI MEP latency	Motor deficit	0.43	**0.001**	0.21
Left FDI CMCT	Motor deficit	0.40	**0.004**	0.08
Right TA MEP amplitude	Sex	−0.32	**0.017**	0.18
	Motor deficit	−0.37	**0.005**	
Right TA MEP latency	Motor deficit	0.66	**<0.001**	0.39
	Sex	0.23	**0.042**	
Right TA CMCT	Motor deficit	0.61	**<0.001**	0.31
Left TA MEP amplitude	Motor deficit	−0.38	**0.006**	0.12
Left TA MEP latency	Motor deficit	0.63	**<0.001**	0.36
Left TA CMCT	Motor deficit	0.58	**<0.001**	0.30

*Legend:* FDI = first dorsal interosseous muscle; TA = tibialis anterior muscle; MEP = motor-evoked potential; CMCT = central motor conduction time; numbers in bold = statistically significant *p* values.
